# Seroprevalence and Molecular Detection of Brucellosis in Hospitalized Patients in Lahore Hospitals, Pakistan

**DOI:** 10.3390/idr13010018

**Published:** 2021-02-08

**Authors:** Riffat Yousaf, Iahtasham Khan, Wasim Shehzad, Riaz Hussain, Shahzad Ali, Heinrich Neubauer, Gamal Wareth

**Affiliations:** 1 Institute of Biochemistry & Biotechnology, University of Veterinary and Animal Sciences Lahore, Lahore 54000, Pakistan; riffatyousaf.biotch@gmail.com (R.Y.); wasim.shehzad@uvas.edu.pk (W.S.); 2 Section of Epidemiology and Public Health, University of Veterinary and Animal Sciences, Lahore Sub-Campus Jhang, Lahore 35200, Pakistan; 3 Department of Pathology, Faculty of Veterinary and Animal Sciences, The Islamia University of Bahawalpur, Bahawalpur 63100, Pakistan; dr.riaz.hussain@iub.edu.pk; 4 Wildlife Epidemiology and Molecular Microbiology Laboratory (One Health Research Group), Discipline of Zoology, Department of Wildlife and Ecology, University of Veterinary and Animal Sciences, Lahore 35200, Pakistan; shahzad.ali@uvas.edu.pk; 5 Friedrich-Loeffler-Institut, Institute of Bacterial Infections and Zoonoses, Naumburger Str. 96a, 07743 Jena, Germany; heinrich.neubauer@fli.de

**Keywords:** brucellosis, hospital patients, RBPT, RT-PCR, Lahore

## Abstract

Brucellosis is one of the most notorious zoonoses worldwide. The disease is common and endemic in humans and animals of Pakistan, but lack of awareness and lack of research have resulted in an increased incidence in the human population. The present study aimed to determine the seroprevalence and at molecular detection of brucellosis in patients with clinical symptoms in six different hospitals from Lahore, which is the capital city of Punjab province. A total of 218 blood samples were collected from hospitalized patients. The samples were initially screened by the Rose Bengal Plate Test (RBPT), and then quantitative real-time PCR (qRT-PCR) was applied. An overall seroprevalence of 17% (37/218) was found. The highest prevalence was found at the Lady Health center (36.53%), which was followed by the Lady Willingdon Hospital (28.6%). Female patients showed a higher seroprevalence than males and peaked at 34% (n = 32) for women who suffered from abortion. In total, 16.8% of patients younger than 30 years showed seropositive reactions, while the prevalence was 19% in patients between 31 and 50. Thirty-three DNA samples from 24 seropositive and nine seronegative patients tested positive, 32 samples were found positive for *B. abortus* DNA, and one sample failed to be identified at the species level. Almost all positive cases had direct contact with animals and consumed unpasteurized dairy products. Research on human brucellosis is still scarce in Pakistan. For the diagnosis of brucellosis, serology and molecular tools should be combined if isolation by culture is not possible. Nationwide control activities and increasing awareness for zoonotic brucellosis are needed.

## 1. Introduction

Brucellosis is one of the most common zoonotic diseases worldwide. It is affecting both animals and humans and caused by the members of the genus *Brucella* (*B.*) [1]. Among twelve known *Brucella* spp., only *B. melitensis, B. abortus, B. canis,* and *B. suis* (except biovar 2) are associated with human infection [2]. Brucellosis is an occupational disease affecting mostly people in contact with animals or animal products, particularly in agricultural countries where most of the population is involved in livestock farming and land cultivation. The clinical symptoms of human brucellosis are non-specific and can be mistaken for other diseases causing fever. Infection in humans is mainly associated with undulant fever, severe back pain, headache, joint pain, weakness, weight loss, loss of appetite, depression, and night sweating [3]. Arthritis, orchitis, endocarditis, hepatomegaly, and splenomegaly have been seen in patients who suffered from brucellosis [4,5]. Brucellosis is transmitted from diseased animals to humans via infected fluids and discharges such as blood, vaginal secretions, placental fluids, and aborted fetus [6]. The bacterial transmission from person to person is rare, but sexual contact and breast-feeding have been proven to transmit brucellae [7]. Common routes of infection include direct inoculation through cuts, inoculation through the eyes’ conjunctival sac, abrasions of the skin, and inhaling of infectious aerosols [8,9]. Occupational exposure to animals and the consumption of unpasteurized milk and dairy products such as soft cheese and yogurt are the most common risk factors for human brucellosis [10].

Brucellosis poses significant public health challenges, particularly in developing countries of the Middle East and the Mediterranean basin [11,12]. In Pakistan, the incidence of brucellosis is increasing day by day due to a lack of awareness [13]. Few studies concerning human brucellosis in Pakistan have been carried out in the last decade [14,15,16,17,18]. The present study aims to determine the seroprevalence of brucellosis and molecular detection of *Brucella* spp. by using PCR in patients with fever and general non-specific clinical symptoms, which can be caused by a variety of health conditions, from six different hospitals in the district of Lahore, the provincial capital of Punjab, Pakistan.

## 2. Materials and Methods

### 2.1. Sample Collection and Serology

The seroprevalence of brucellosis was investigated, involving 218 patients with fever of unknown origin (FUO) and general non-specific clinical signs caused by several diseases, who were consecutively admitted to six public hospitals in Lahore, the capital city of Punjab province of Pakistan from December 2014 to January 2015. The patients were mostly suffering from fever, severe back and joint pain, headache, weakness, loss of appetite, and depression. Samples were collected from eighty-two male patients and 136 female patients; among them, 94 women had a history of abortion. From each enrolled patient, 5 mL of blood was collected and stored at 4 °C. Fifty-two, 48, 42, 36, 20, and 20 samples came from the Lady Health center, the General Hospital of Lahore, Lady Willingdon Hospital, the Jinah Hospital, the Mansurah Hospital, and the Nawaz Sharif Hospital, respectively. According to standard procedures, all blood samples were screened by Rose Bengal Plate Test (RBPT) [19]. Sera were considered positive when showing any degree of agglutination in the RBPT.

### 2.2. Quantitative Real-Time Polymerase Chain Reaction (qRT-PCR) Assay

DNA was extracted from serum samples using FavorPrep^TM^Blood/Cultured Cell Genomic DNA Extraction Mini Kit. All extracted DNA was examined by a *Brucella* genus-specific and species-specific (*B. abortus* and *B. melitensis*) qRT-PCR assay according to Probert et al. [20]. Amplification of gene and real-time fluorescence detection was performed on CFX96^TM^ Real-Time PCR System (BIO-RAD C1000 Touch^TM^ Thermal Cycler) (Bio-Rad, Foster-city, California, USA). The reactions were performed in duplicate using reference *B. melitensis* and *B. abortus* DNA as positive controls and HPLC nuclease-free water as a negative control. Only positive *Brucella* genus-specific samples were subjected to species-specific (*B. abortus* and *B. melitensis*) multiplex qRT-PCR.

### 2.3. Statistical Analysis, Questionnaire, and Ethical Approval

The Chi-square test was done using the statistical software SPSS version 17.0. A structured questionnaire was developed for the personal record containing demographic data (age, sex, and residency), clinical manifestations, profession, history of exposure to animals, and milk consumption habits of patients. This study was approved by the ethical committee of the University of Veterinary and Animal Sciences Lahore, Pakistan. Verbal and written permission was taken from each patient before collecting the blood sample.

## 3. Results and Discussion

Brucellosis is a common and endemic disease among animals and humans in Pakistan; however, this disease is still neglected in human medicine, and nationwide control activities and surveillance programs have never been structured [21]. Among 218 blood samples collected from hospitalized patients in different hospitals of Lahore, Pakistan, the overall RBPT seroprevalence of brucellosis was 17% (37/218) (95% Confidence Interval (CI): 11.9–21.9). The highest prevalence was seen in the Lady Health center (36.53%), followed by the Lady Willingdon Hospital (28.6%). Only 4/48 (8.33%) and 2/36 (5.55%) of patients from the General Hospital and Jinah Hospital were seropositive, respectively. All samples collected from the Mansurah (n = 20) and Nawaz Sharif (n = 20) Hospitals were seronegative. Two out of 82 male patients were seropositive, while the female patients showed 34% higher seroprevalence (n = 32) in women who suffered from abortion and 7.14% in patients with clinical signs, e.g., fever, weakness, headache, abdominal and joint pain, and loss of appetite. Out of all patients, 16.8% of patients younger than 30 years were seropositive, while the prevalence was 19% in patients between 31 and 50 (Table 1). In general, few studies focusing on brucellosis in humans in Pakistan were carried out. Moreover, studies included women with a history of abortions and clinical signs are scant in Pakistan. Thus, this study provides new data and knowledge to scientists and readers about human brucellosis in Pakistan.

DNA extracted from serum samples has been tested initially by a *Brucella* genus-specific bcsp31 PCR. Out of 37 seropositive samples, 24 samples were PCR positive for *Brucella* and 13 were negative. On the other hand, 181 samples showed no agglutination and were considered seronegative. Hence, nine samples were PCR positive for genus *Brucella*. All genus PCR (n = 33) positive samples were tested by species-specific IS711 (*B. abortus* and *B. melitensis*) qRT-PCR, and 32 samples were found positive for *B. abortus* DNA, and one failed to be identified at the species level (Table 2). Therefore, using broad-range PCR techniques, including other *Brucella* species in the investigation of brucellosis, is important to be considered for future studies. These results showed that quantitative real-time PCR assay could effectively amend RBPT. Seronegative/culture-positive results have been recognized in animals [22], resulting in the maintenance and spread of infection. Thus, the diagnosis of brucellosis has to be accompanied by molecular detection and/or culture in endemic countries in humans and animals.

Although brucellosis has been extensively investigated in farm animals in Punjab [23,24,25,26], this disease in humans is not well investigated yet. Few studies were carried out relying on serology and PCR, while studies using/applying culture and subsequent identification are next to none. Out of 360 serum samples collected from workers at four slaughterhouses of the Lahore district, 21.7% were positive by ELISA [14]. In Malakand, Khyber Pakhtunkhwa province, a seroprevalence of 27% was found in 304 female patients from a high-risk population using ELISA [18]. Age-wise prevalence was higher (32.25%) in females 21–30 years old. In the same district, the seroprevalence was 32.90% among hospitalized patients in Peshawar, which is the largest and capital city of Khyber Pakhtunkhwa province [27]. Of the 429 serum samples collected from pregnant women at the Benazir Bhutto Hospital, Rawalpindi, 5.8% were seropositive [16]. In a study group of 70 patients at the Teaching Hospital of Abbottabad, 49 patients were seropositive. The majority of patients (n = 35) were between 21 and 40 years [28]. At Rawalpindi and Islamabad hospitals, 10.1% and 5.8% of acute febrile patients were tested positive by RBPT and *B. abortus* PCR, respectively [17].

Examination of 262 serum samples collected from different occupational personnel in the Potohar plateau of northeastern Pakistan showed 6.9% seropositive samples. All patients were confirmed to be infected with *B. abortus* by RT-PCR [15]. The prevalence was higher in people consuming raw dairy products. The detected low prevalence of 6.9% on the Potohar plateau of northeastern Pakistan [14] and 9.33% in Azad Jammu and Kashmir [29] might be explained by a smaller chance of animal contacts and reduced intake of unpasteurized milk in these areas. The different serological tools used could also be a reason for different prevalences due to differences in each test sensitivity and specificity.

Different serological and molecular methods have been extensively used to diagnose brucellosis. However, quantitative real-time PCR is a very reliable and specific technique for rapidly detecting and differentiating *Brucella* species in samples when a positive result is gained. The qRT-PCR test results are significant in hospitalized patients with clinical signs and negative RBPT results, allowing the early and rapid confirmation of brucellosis in humans.

In our study, a lower seroprevalence was recorded in males (clinical cases 2.43%) when compared to females. In previous studies, higher seroprevalences rate were recorded in women (37%) than in men (24.2%) in hospitalized patients of Peshawar [27]. The higher seroprevalence rates in females are caused because the housewives and female workers in rural areas have more direct contact with their livestock because of their daily duties. Sometimes, they help animals at birth and remove aborted fetuses without any precautionary measures. As brucellosis is an occupational disease, persons of this age group (≤50) are at risk because of prolonged exposure to infected animals. Therefore, activities in areas where animal brucellosis is not controlled increase the risk of acquiring brucellosis.

Assessment of the awareness for brucellosis among the personnel of small dairy farms was conducted in Punjab in 2015. The study revealed that 97% of farmers have no knowledge about brucellosis and its transmission routes. Additionally, 66% of the farmers’ families consumed raw milk and dairy products [13]. Cattle and buffaloes are the primary sources of milk in Pakistan, and *B. abortus* is the predominant cause of bovine brucellosis. This explains the presence of *B. abortus* in all of the human cases in this study. Seroprevalence of bovine brucellosis was investigated in 420 herds in seven districts of Pakistan, and the herd prevalence was 16.2% [29]. In Punjab, the seroprevalence was 3.9% and 3.3% by RBPT and ELISA, respectively, at institutional-owned livestock farms, and only DNA of *B. abortus* was amplified from the seropositive samples [26]. Some risk factors for brucellosis were researched from the patients of this study. Most of the patients lived in rural areas, and various were involved in livestock farming. Most of these patients had direct contact with their animals during their daily routine and consumed unpasteurized milk and milk products. Most patients diagnosed with fever were treated as dengue or malaria or uncomplicated fever patients. Confirmed brucellosis cases were treated according to the World Health Organization (WHO) protocol; however, the patients’ treatment history was not mentioned because self-medication and treatment without prescription are widely implemented in developing countries, including Pakistan. There was no clear information available from patients to be included in the current study as a control group e.g., patients with different but precise etiological diagnoses such as autoimmunity, dengue, and malaria, which are confused with brucellosis in the differential diagnosis.

## 4. Conclusions

In conclusion, brucellosis is a significant public health issue in different hospitals in the Lahore area, especially in pregnant women. The disease can be controlled in animals by using preventive measures e.g., hygiene and vaccination. Still, the consumption of unpasteurized dairy products is a significant risk factor for infection. Regular screening of animals and owners has to be implemented in Pakistan’s endemic areas. Increased awareness of risk factors, method of transmission, and importance of biosafety during handling of infected or aborted animals is required among occupational personnel e.g., veterinarians, abattoir workers, farmers, and animal holders in contact with animals. The government public health authorities should be aware of the zoonotic importance of brucellosis in the general public, and implementation of a national surveillance program is needed. Diagnosis of brucellosis should combine serology and molecular diagnosis.

## Figures and Tables

**Table 1:** Seroprevalence of brucellosis in 218 patients admitted to six hospitals of the Lahore district of Pakistan using RBPT.

Variable	Category	No. of Patients	RBPT Positive	Prevalence (%)
Hospital	Lady Health center	52	19	36.53
	General Hospital	48	4	8.33
	Lady Willingdon Hospital	42	12	28.6
	Jinah Hospital	36	2	5.55
	Mansurah Hospital	20	0	0.00
	Nawaz Sharif Hospital	20	0	0.00
Total		218	37	17
Gender	Male (clinical cases)	82	2	2.43
	females with a previous history of abortion	94	32	34.0
	Female with clinical signs and without a history of abortion	42	3	7.14
Age, years	≤30	143	24	16.8
	31–50	68	13	19
	≥51	7	0	0.00

**Table 2:** The relation between results of serology and molecular diagnostics and age of patients in samples collected from 218 fever of unknown origin (FUO) patients from six different hospitals in the Lahore district of Pakistan.

Clinical Cases				
	≤30 Years	31–50 Years	>50 Years	Total
Male tested	58	19	5	82
RBPT + ve	1	1	0	2
qRT-PCR + ve	1	1	0	2
Female tested	23	17	2	42
RBPT + ve	3	0	0	3
qRT-PCR + ve	3	3	1	7
Abortion cases				
Tested cases	62	32	0	94
RBPT + ve	18	14	0	32
qRT-PCR + ve	14	10	0	24

## Data Availability

Not applicable.
